# Optical Temperature Measurement in Unsteady Plasma Free Jet

**DOI:** 10.1088/1361-6501/ad24b7

**Published:** 2024-02-19

**Authors:** Tobias Hermann, Eric Won Keun Chang

**Affiliations:** Oxford Thermofluids Institute, University of Oxford, Southwell Building, Osney Mead, Oxford OX2 0ES, UK

## Abstract

An Argon plasma free jet is investigated using spectrally narrow bandpass filtered high-speed imaging. The images were captured at 16kHz with an exposure time of 3.9 μs and then calibrated for absolute radiance. The free jet exhibited behaviour consistent with turbulent free shear flow and maintains an axisymmetric shape. Significant local fluctuations were observed over time, growing in strength and size as the flow convected downstream. Assuming local thermodynamic equilibrium and self-similar free-jet temperature profiles, the flow radiance is used to determine the local plasma temperature and the jet width. Then, both steady and unsteady flow models were applied to account for the jet fluctuation. In regions of low fluctuations near the nozzle exit, both models show good agreement for centreline temperatures, measuring approximately 11,200K. In regions of significant fluctuations, the assumption of steady flow leads to an overestimation of 32% for temperature, 18% for jet width, and 41% for total jet power. The unsteady analysis approach results in lower temperatures and smaller jet widths while simultaneously satisfying momentum and energy conservation.

## 1 Introduction

Spacecraft entering an atmosphere are exposed to extreme surface heating due to the conversion of kinetic energy into internal energy of the ensuing flowfield as they pass through the atmospheric gas hull [1]. Heat shields are used to protect payload or crew from the severe heat loads [2]. For the purpose of validating the heat shield material performance using a ground testing methodology, a thermochemical load similar to the flight case has to be imparted onto the materials’ surface [3]. Arcjet wind tunnels are widely utilised in the experimental spacecraft entry community, primarily for validation of heat shield materials and for conducting aerothermodynamic research [4, 5, 6, 7, 8, 9, 10]. Flows created by arc-jets simulate the high-enthalpy plasma flow in the region between the compression shock and the surface of a re-entry vehicle. [11]. This flow is generated by passing test gas through an electric arc formed by an axisymmetric arrangement of a central cathode within a hollow anode. The gas is turned into a plasma state by heat addition via the electric arc and is then expanded through a nozzle into a test chamber. Within the test chamber, the flow can be interrogated with different diagnostic measurement techniques [12, 13], and material samples are tested [14].

Due to the complex generation of the plasma flow within an arc-jet and uncertainties in numerical models, achieving an accurate simulation of the freestream is challenging. Therefore, experimental freestream characterisation is essential to improve our understanding of plasma flows. As heat shield material response and the flow condition extrapolation from ground to flight depend significantly on the freestream flow properties, accurate knowledge of these properties is essential for improving thermophysical models and to increase confidence in boundary conditions for high-temperature material investigations. As such, measurement technology for plasma flows is an essential component of the ground testing methodology. Optical methods have gained prevalence due to their non-intrusive nature, potential high resolution imaging capabilities, and the recent rapid improvement in commercially available camera and laser technology [15]. Analysing spontaneous emission of plasma radiation has been widely used to determine plasma temperature and enthalpy [16, 17]. Common analysis methods include Boltzmann plots [18], spectral shape fitting [19], and using absolute radiance [20] to infer these properties. Due to the limited sensitivity of sensors, such as CCD cameras, the optical integration time for emission measurements is often in the order of seconds. Contrary to these relatively long-exposure measurements, modern high-speed cameras have been employed to capture transient measurements of plasma flows, providing insights into the temporal evolution of radiative emission [21, 22, 23, 24]. Hermann et al. and Zander et al. show that a high enthalpy arc-jet freestream can be highly transient on the order of several kHz, consisting of individual plume structures considerably smaller than the time-averaged plasma jet appearance [12, 21]. These transient characteristics likely originate from a change in arc-location over time, and from turbulent fluctuations in the free jet shear layer [25]. Furthermore, the flow temperature in an inductively coupled plasma facility can change by as much as 4,000 K over time, with a mean temperature of approximately 13,400 K [22].

**Figure 1:**
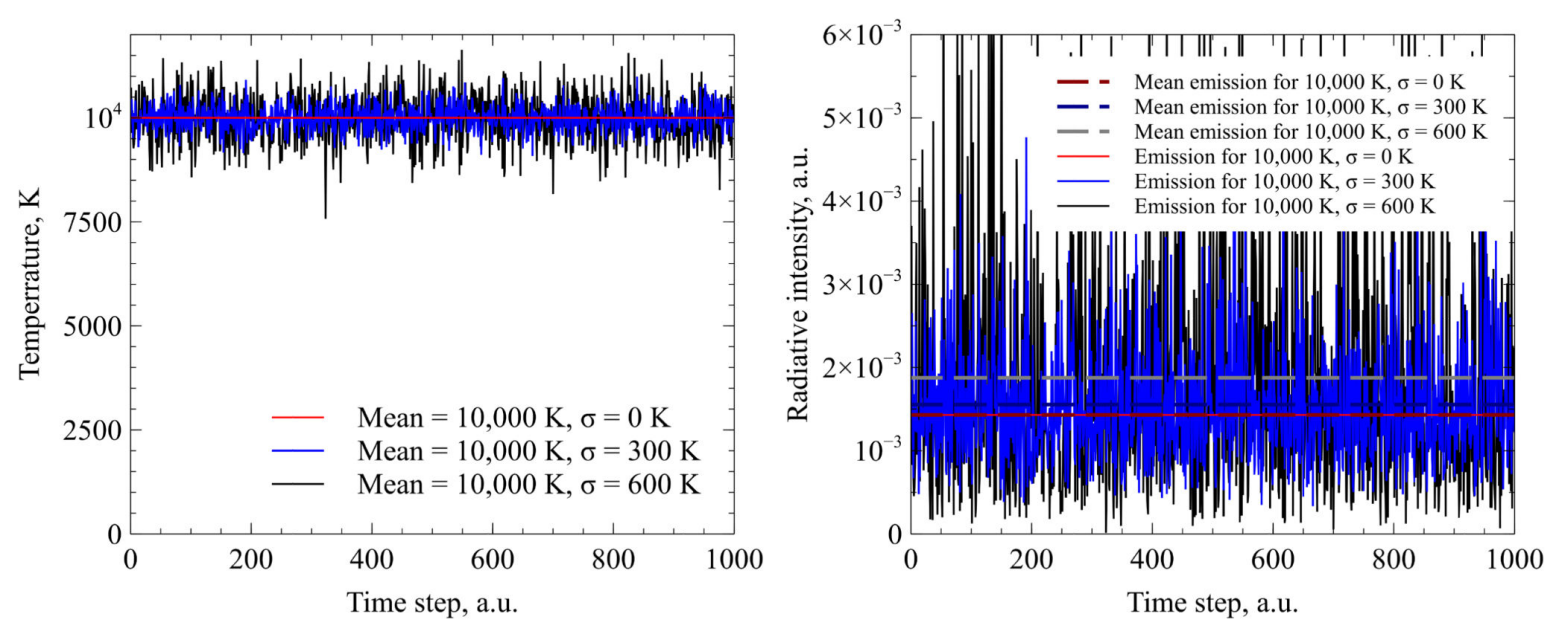
Artificial temperature fluctuation over time (left) with respective radiative emission fluctuation of the 706.72 nm line over time and their temporal averages (right).

These findings pose a challenge for optical methods that capture plasma radiation over time periods much larger than the fluctuation frequency. In such cases, the recorded radiation will represent a temporal average over the exposure time. However, as will be shown in this paper, a temporal average of emitted radiation is not a unique function of the temporally averaged temperature. Since the intensity of emitted radiation depends non-linearly on the plasma temperature, the temporal average depends on the amplitude of fluctuations and their probability distribution. To illustrate this point, artificial data has been generated for three different cases: A pure Argon plasma is simulated with a mean temperature of 10,000 K which is subject to Gaussian distributed fluctuations of *σ* = 0, 300, 600 K where *σ* represents the standard deviation. Assuming the Argon plasma in local thermodynamic equilibrium, the instantaneous radiative emission intensity of the atomic transition line at 706.72 nm has been calculated based on the temperature at each time step. The local radiative emission coefficient of the line is calculated assuming a Boltzmann distribution of excited electronic states. The temperature and resulting emission data is shown in Fig. 1. The right hand side plot also contains the temporal average of the emission intensity for each case. For fluctuations with *σ* = 300 K, the average emission is 9% higher than for a steady state case, and for *σ* = 600 K, it is 31% higher. Even though all three cases feature the same mean temperature, significant differences in the average radiation exist.

For a steady state case with a given set of thermodynamic properties the intensity of a radiative transition is unique. However, as shown above, this is not the case if significant fluctuations exist. As previously mentioned, the majority of emission spectroscopy setups do not capture the transient evolution of plasma radiation but instead rely on temporally averaged measurements. Based on these data, the inferred thermodynamic quantities, such as temperature and enthalpy, may be affected by errors brought about by the unsteadiness in the plasma flow. This arises from the absence of a unique relationship between emission intensity and average temperature if temporal fluctuations are unknown. This problem has motivated the current study to experimentally characterise the unsteadiness of a plasma flow to quantify and correct the measurement bias introduced by unsteady flow behaviour.

In this study, an Argon plasma free jet is investigated using spectrally narrowband filtered high-speed imaging. The miniaturised arc-jet facility OPG1 at the University of Oxford is utilised to generate an Argon free-jet in a vacuum chamber. Details of the experimental setup are provided in section 2, experimental measurement results are given in section 3, the analysis methodology is described in section 4 with the analysis results given in section 5. Finally, the implications of unsteadiness on measured plasma plume properties are discussed in section 6, and section 7 summarises the work.

## 2 Experimental setup

This section describes the test facility and employed diagnostic measurement techniques. A combination of optical and probe-based measurements is used to investigate an Argon plasma free jet.

### 2.1 Miniaturised arc-jet facility OPG1

**Figure 2:**
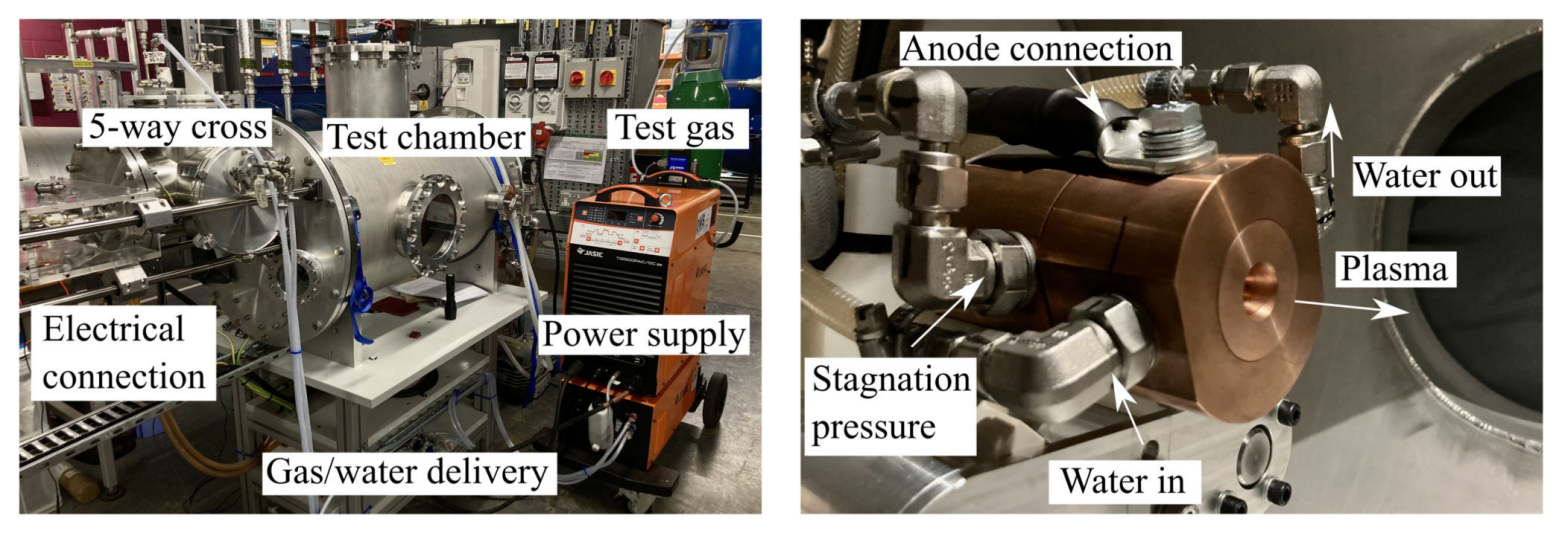
OPG1 small-scale plasma wind tunnel facility overview (left), and plasma generator inside testing chamber (right) [28].

**Figure 3:**
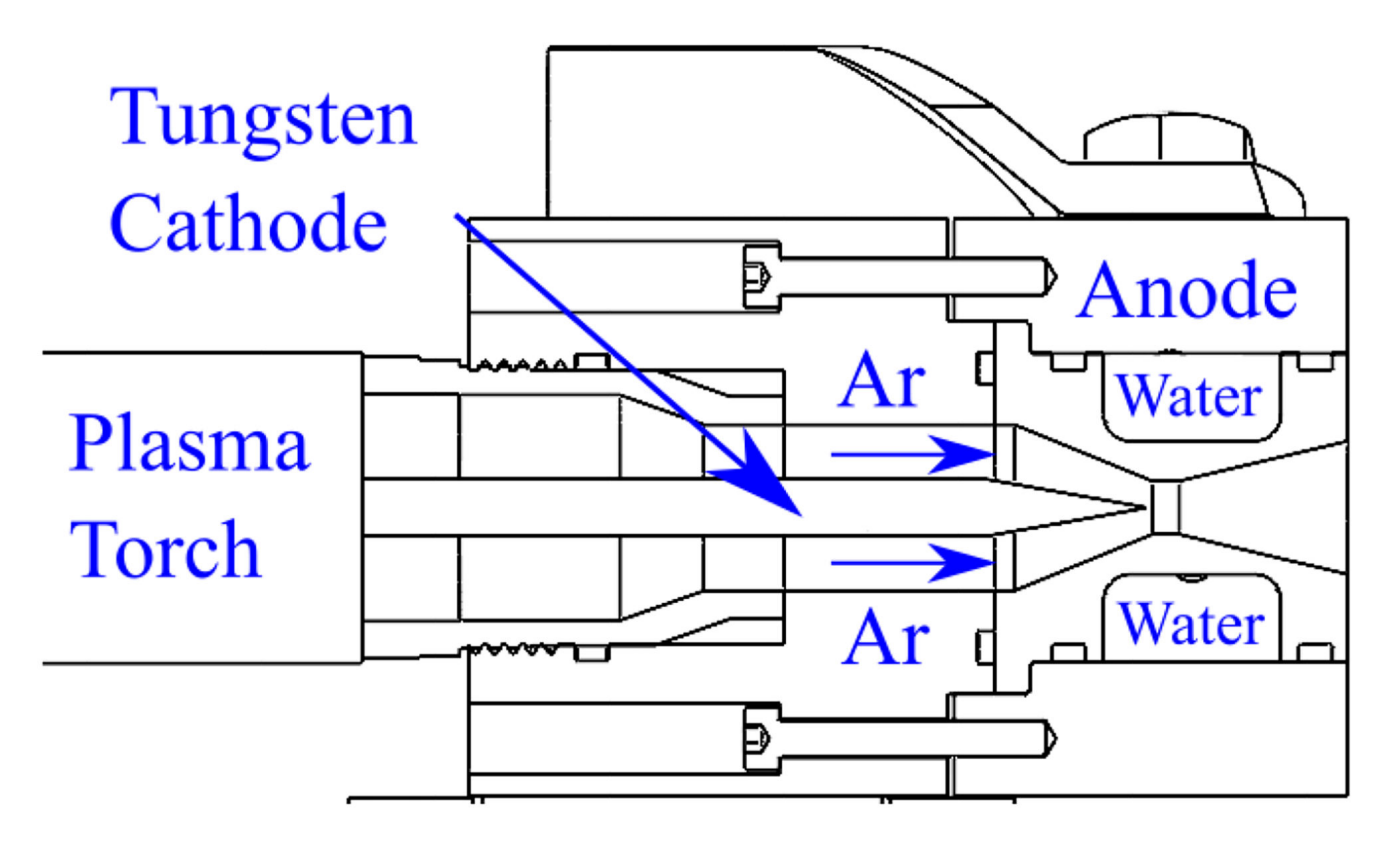
Cross-sectional view of plasma generator [27].

Figure 2 displays a photograph of the OPG1 miniaturised plasma wind tunnel facility, which consists of a thermal arc-jet plasma generator, an electrical power supply, a gas/water delivery system, a vacuum exhaust system with heat exchanger, a model mounting system, and a data acquisition system. The electrical power for the arc-jet is supplied by a Jasic TIG 500P AC/DC tungsten inert gas (TIG) welding power supply, which can generate up to 500A of current and up to 21.5kW of power [26]. The plasma generator utilises a tungsten cathode with 2% thorium and a water-cooled copper nozzle as the anode. Argon is passed through an electric arc generated between the electrodes which elevates its temperature to several thousand Kelvin [27]. The schematic of the generator architecture is shown in Fig. 3.

OPG1 was commissioned in 2023 and is a miniaturised stand-alone facility capable of performing high-temperature material testing and fundamental experiments on plasma flows. It is further developed to serve as a pre-heating device for sub-scaled models in hypersonic impulse facilities [28]. The plasma generator shown in Fig. 2 is located inside the test chamber. The nozzle throat diameter is 6 mm and expands to 15 mm at the exit. Multiple water-cooling lines are in place to maintain the arc-jet assembly temperatures below critical values inside the vacuum chamber. The facility is equipped with various optical and probe diagnostics including a slug-calorimeter, a pitot pressure probe, narrow bandpass filtered imaging using high speed cameras, and optical emission spectroscopy. Facility conditions are measured including test gas mass flow rate using an Omega FMA5527A mass flow controller, test section and stagnation chamber pressure, electrical current and voltage, and cooling water heat loss. The flow condition used featured an arc current of 150 A, and a voltage of 20.1 V. The argon mass flow rate was set to 0.3 g/s and the test chamber pressure was at 46 mbar during the acquisition of optical data.

### 2.2 Filtered high-speed imaging

The plasma radiation was imaged by a Photron Fastcam Mini UX 100 800K-M-4G high speed camera at a frame rate of 16 kHz and with an exposure time of 3.9 *µ*s. The camera was equipped with a 70 mm - 200 mm Tamron zoom lens featuring a F-number of 2.8. The camera provides a full image resolution of 1280*×*312 pixels with a bit depth of 12. The camera captured the flowfield exiting the nozzle and forming a free-jet. The high speed camera was equipped with a narrow-bandpass filter of centre wavelength 700 nm with 10 nm full width half maximum spectral width. The camera viewed the plasma through a BK7 window and was located 430 mm laterally from the plasma-jet axis of symmetry and 20 mm downstream of the nozzle exit plane. It was oriented to be perpendicular to the plasma flow direction.

**Figure 5:**
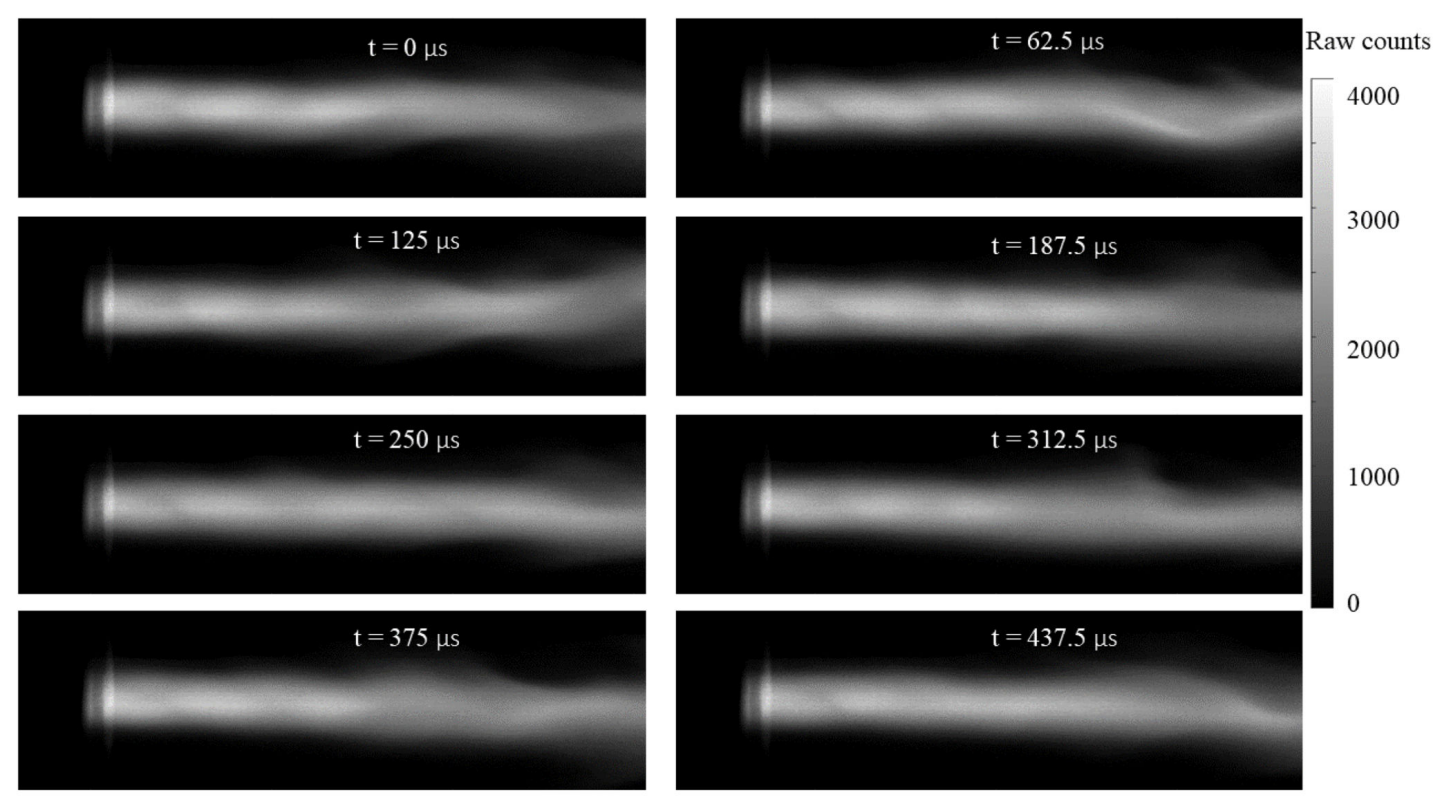
Image sequence of plasma flow. The plasma flows from left to right exiting the nozzle into the evacuated test chamber. Each image is separated by 62.5 *µ*s with an exposure time of 3.9 *µ*s.

An example sequence of consecutive images is shown in Fig. 5. In this figure, the nozzle is on the left of each image and the flow direction is from left to right. It is evident that the instantaneous jet appearance changes from image to image. However, the relative amount of change increases downstream as the jet becomes increasingly dominated by turbulent structures originating from the shear layer between cold gas in the test chamber and the hot plasma [25].

**Figure 6:**
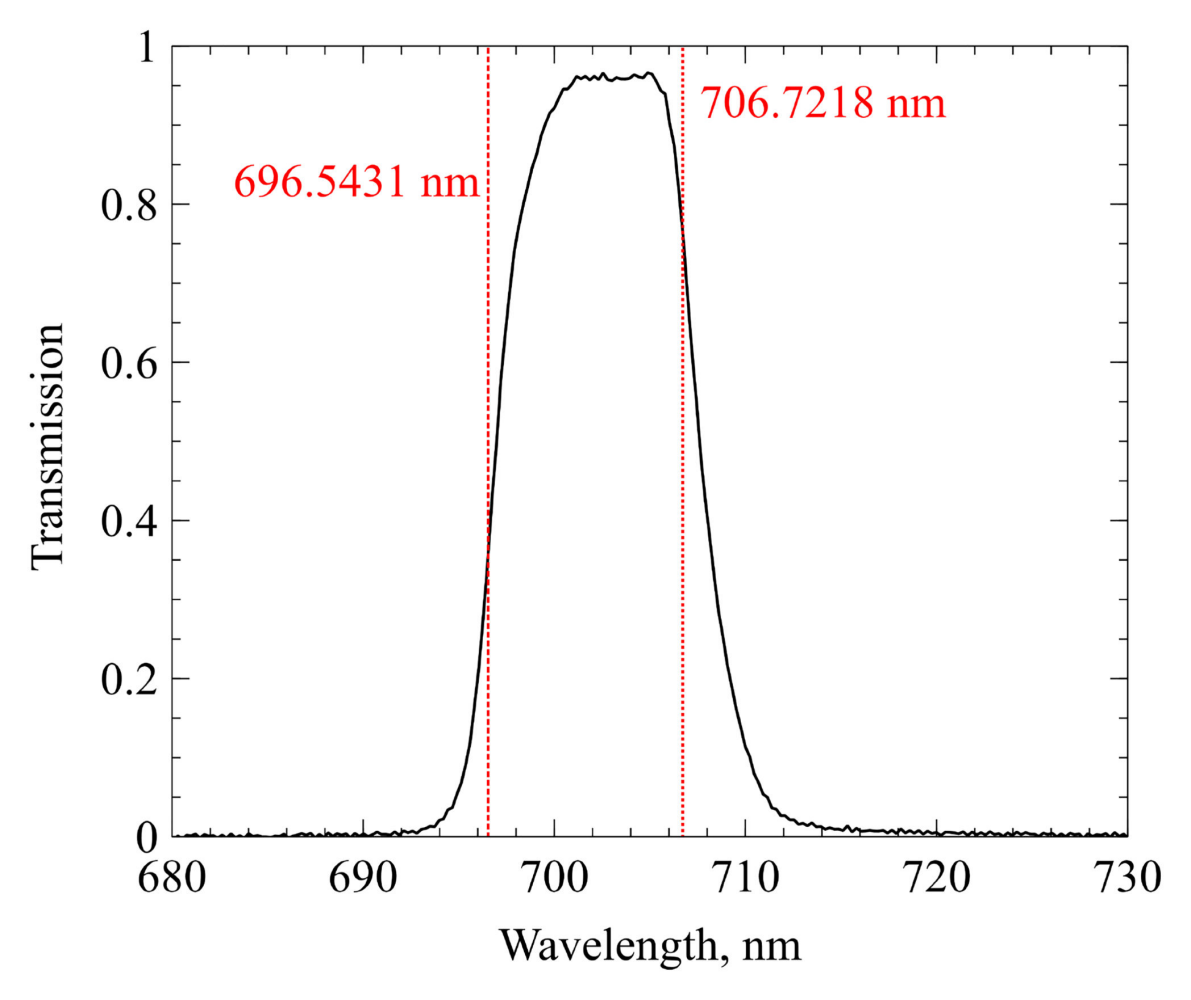
Transmission spectrum of optical filter with two Argon line locations.

The transmission spectrum of the optical filter has been measured using a fibre-coupled Thorlabs CCS200 spectrometer and a Bentham Instruments SRS8 integrating sphere. The spectrometer was exposed to the integrating sphere’s radiation both with and without the optical filter. The two background-corrected datasets were divided by each other to obtain the transmission at each recorded wavelength with a spectral resolution of 0.21 nm. The resulting data is show in Fig. 6. The figure also contains two lines indicating the central wavelength of two prominent Argon lines that fall within the transmissive regime of the filter. These two lines are the only major contributors to the radiation recorded through the filter, as their Einstein coefficients are larger, and their upper state excitation energy is significantly lower than those of neighbouring lines. Assuming a Boltzmann distribution of excited energy states, this leads to several orders of magnitude more radiation from the two lines at *λ*_1_ =696.54 nm and *λ*_2_ =706.72 nm. The measured transmission value for the centre wavelength of these two lines is *τ*_1_ =0.358 and *τ*_2_ =0.762 respectively.

The optical system consisting of camera, lens, bandpass filter, and test section window has been calibrated in-situ for absolute radiance using a Bentham Instruments SRS8 integrating sphere. The integrating sphere is placed at the nominal plasma generator centreline which is approximately 43 cm from the camera lens. The procedure developed in [22] has been followed which is repeated in the following. The total radiance received by the camera-lens system behind the filter-window combination is calculated by

(1) $$L_{\text{cal}}=\int L_{\lambda ,\text{IS}}\cdot \tau_{\text{Filter}}\text{d}\lambda ,$$

with *L*_*λ*,IS_ denoting the integrating sphere spectral radiance, *τ*_Filter_ denoting the filter transmission and *λ* denoting wavelength. This value is then used to calculate the calibration factor

(2) $$K=\frac{L_{\text{cal}}}{n_{\text{cal}}/t_{\text{cal},\text{exposure}}},$$

with *n*_cal_ denoting measured counts at each pixel, and *t*_cal,exposure_ denoting exposure time. This value is calculated for each pixel and relates the sensitivity of the camera-lens-system to the radiance that exists behind the filter-window combination. To calibrate a plasma flow experiment, the measured *n/t*_exp_ are calibrated by multiplying them with the calibration factor *K*. This corresponds to the total experimental radiance received behind the filter-window-combination. In order to relate this to the radiance emitted by the plasma, the filter transmission at the two discrete wavelengths *λ*_1_ and *λ*_2_ has to be taken into account. Before applying this correction, the relative intensities of the two lines has to be known in order to determine a unique plasma radiation value. Due to their identical degeneracy-weighted Einstein coefficients *A*_*ul*_ *g*_*u*_ = 1.9 *·* 10^7^ s^−1^ and their almost identical upper state energy levels of *E*_*u*,1_ = 107496.4166 cm^−1^ and *E*_*u*,2_ = 107289.7001 cm^−1^, the radiance emitted by each spectral line is assumed to be identical [29]. This has been confirmed numerically by simulating the emission of these two lines using NASA’s NEQAIR program at enthalpy and pressure levels found in the experiments [30]. Using the analysis methods in [20] for the investigated conditions, the line broadening has been found to be smaller than 0.02 nm which is much smaller than the relative change in filter transmissivity over a transmission line. This leads to the assumption that all line radiance is transmitted at the centre wavelength transmission values *τ*_1_ and *τ*_2_ respectively. Using these assumptions, the total plasma radiance from the two investigated lines is calculated by

(3) $$L_{\text{exp}}=\frac{2\cdot K\cdot n_{\text{exp}}/t_{\text{exp},\text{exposure}}}{\tau_{1}+\tau_{2}},$$

with *n*_exp_ denoting measured counts at each pixel, and *t*_exp,exposure_ denoting exposure time during the experiment. The uncertainty of each pixel value is based on shot-noise and is calculated assuming a Poisson distribution of incoming photons [17] leading $\sqrt{n}$, with *n* being the number of counts. This leads to an uncertainty of approximately 2% for the bright centreline radiance.

## 3 Experimental Results

**Figure 7:**
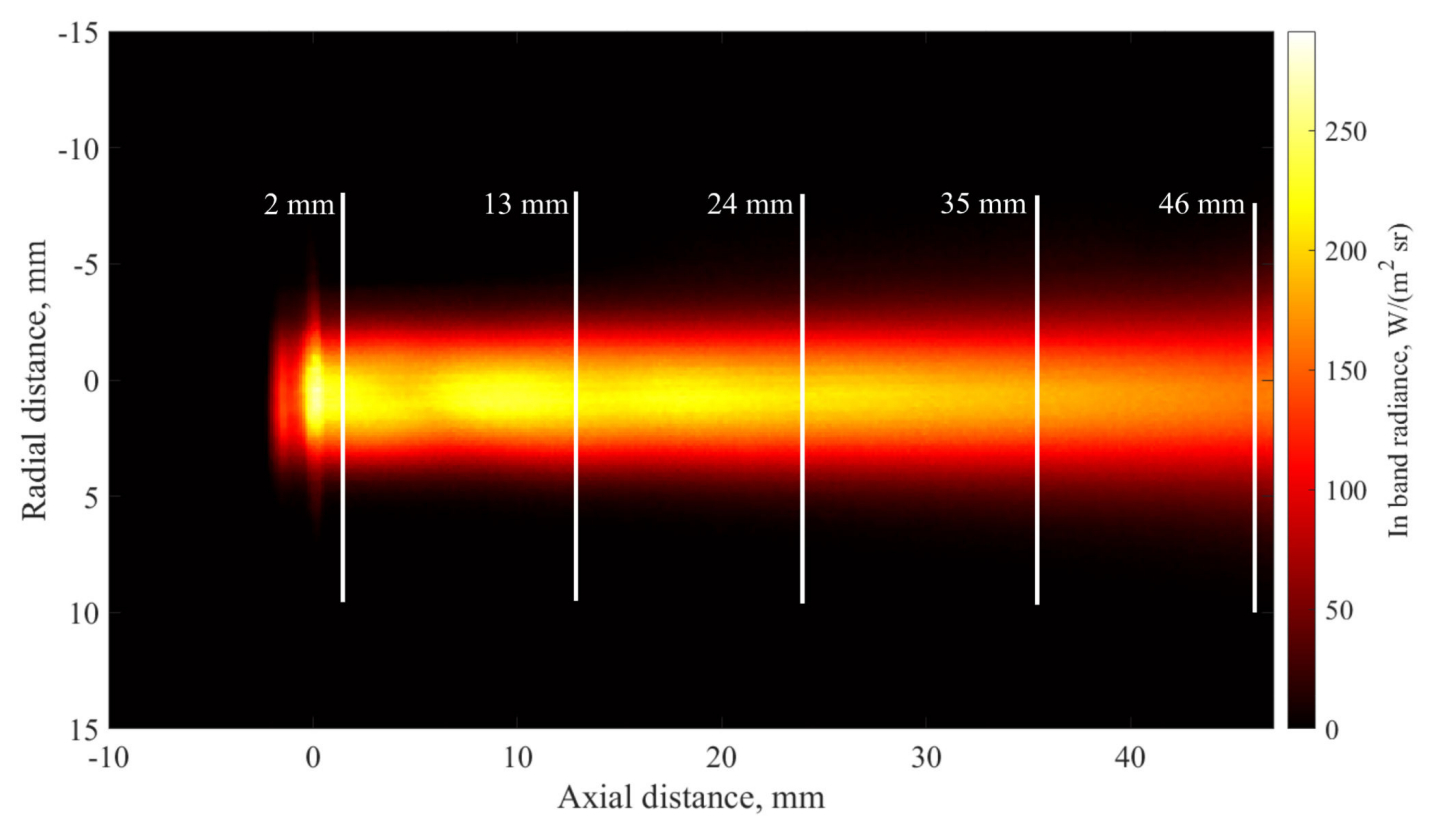
Temporally averaged radiance emitted by the plasma flow within the spectral filter bandpass.

This section presents processed and calibrated data of the measured plasma free jet. Figure 7 displays the temporally averaged emitted radiance of the plasma flow, indicating five locations for each investigated cross-section, positioned at axial distances of 2, 13, 24, 35, and 46 mm from the nozzle exit plane. The image is the result of averaging over 1000 frames which corresponds to a total time of 62.5 ms. The plume has an axisymmetric shape and exhibits the anticipated behaviour of decreasing intensity as the jet progresses downstream. Slight axial changes in emission intensity are visible between 0 mm and 10 mm, which are attributed to a very weak Mach-diamond structure.

**Figure 8:**
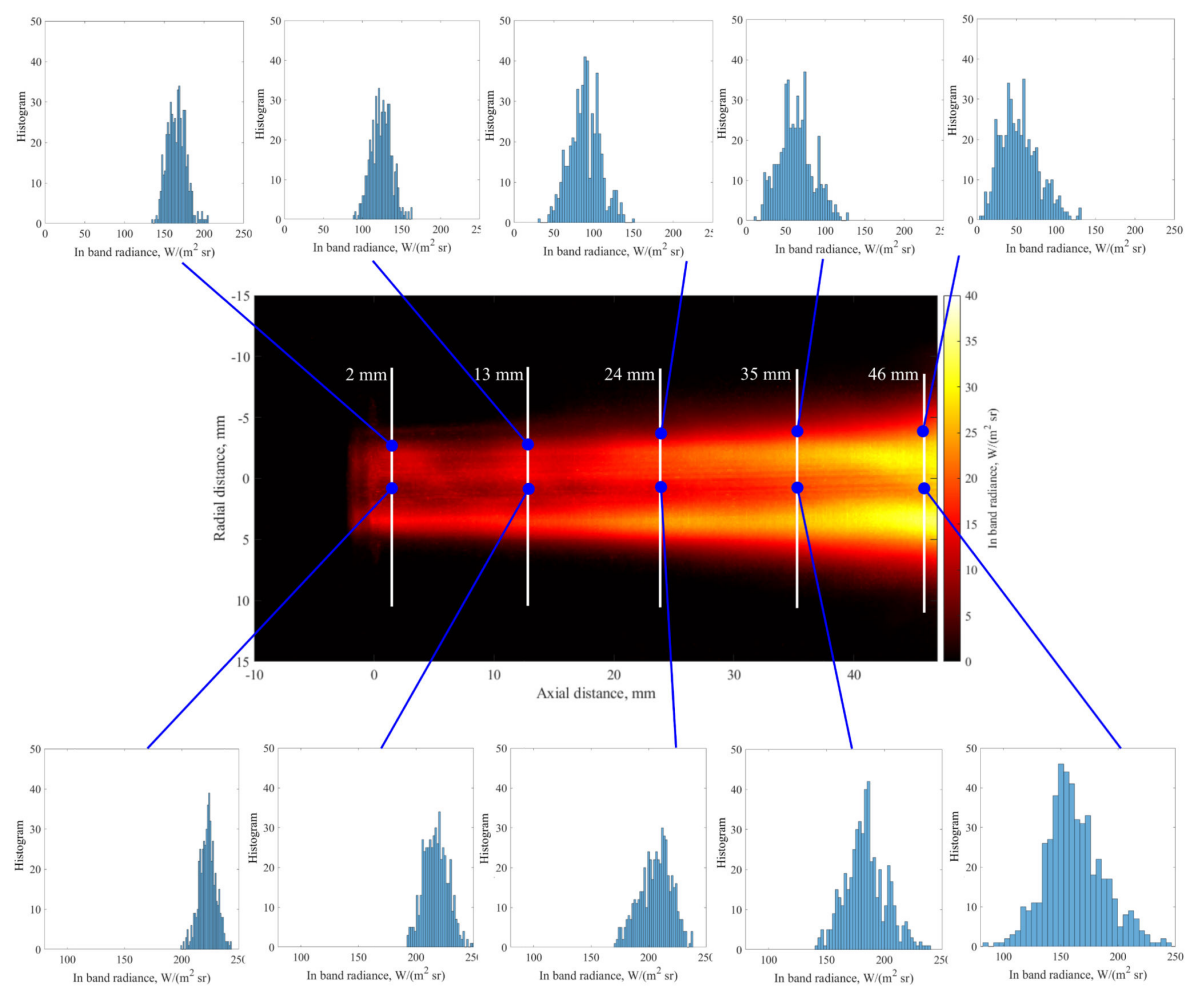
Temporal standard deviation of radiance emitted by the plasma flow within the spectral filter bandpass. Individual **histograms** of emitted in band radiance at select locations.

**Figure 9:**
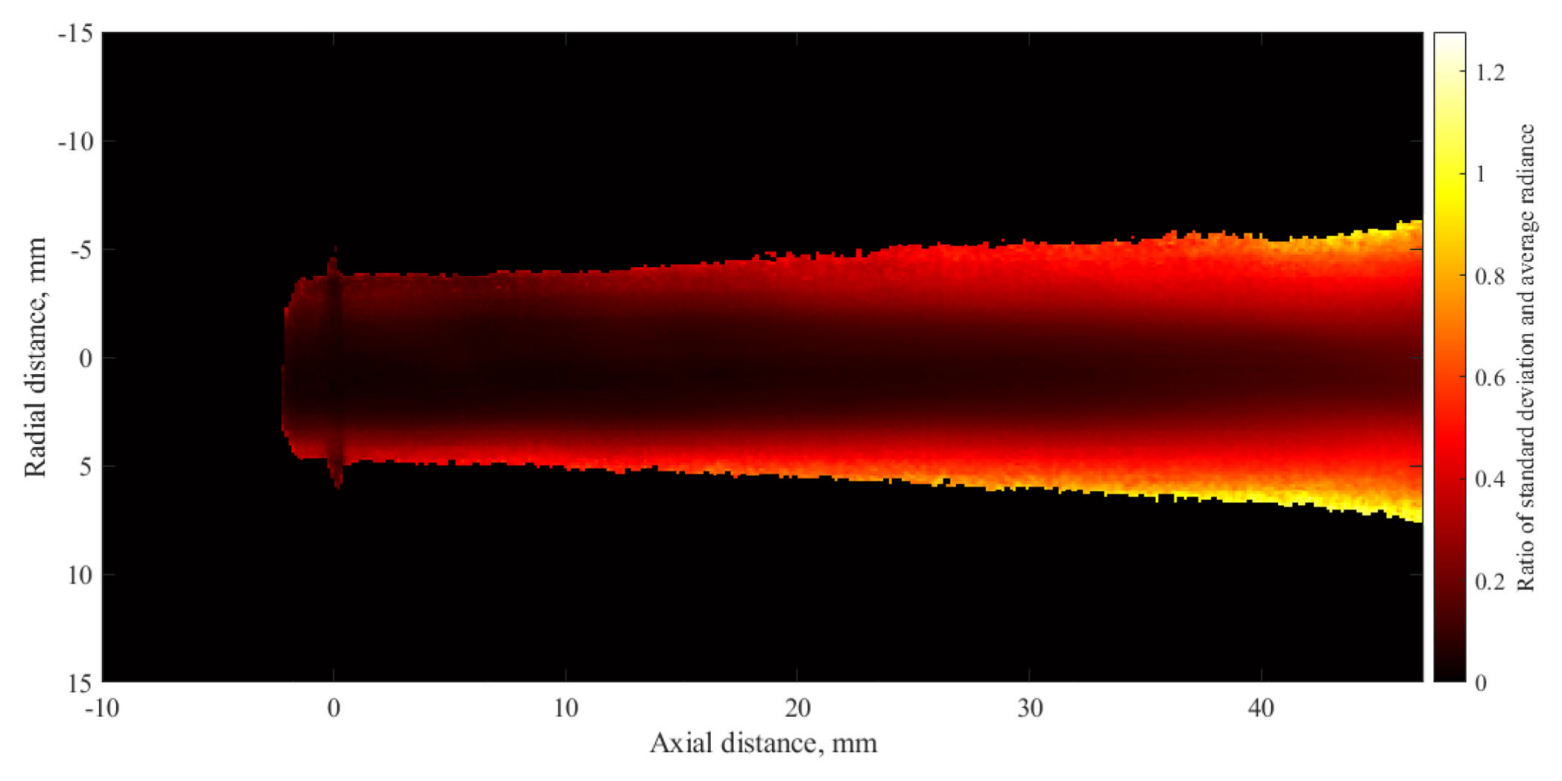
Ratio of temporal standard deviation over average radiation.

Based on the time history of radiance at each pixel, the temporal standard deviation has been calculated and is shown in Figure 8. This image can be interpreted as the strength of fluctuation at each location. Comparing this data with Figure 7, it appears that a double-peak of the fluctuation strength exists at the radial edge of the plume. This means that changes in radiative intensity are comparably small on the centreline of the jet, but reach their maximum value near the edge. Furthermore, the figure shows that fluctuation magnitude increases downstream which is due to the growing scale of turbulent eddies which can also be seen in Figure 5. Furthermore, this image shows that the radial extent of the plume is significantly influenced by fluctuations, as the plume width in Figure 8 is much larger than the average radiance shown in Figure 7. As the magnitude of fluctuations are significant, it can be concluded that especially the plume appearance further away from the centreline is dominated by unsteady effects. This is shown in Figure 9 where the relative strength of standard deviation over average radiance is shown. The calculation has been performed for all pixels which fall within 97 % of the maximum measured radiance. As is clear from this image, the centreline fluctuation is comparatively small, whereas the outer edges of the plume are made up of almost entirely fluctuating emission as the ratio shown tends towards one.

Figure 8 further shows the histogram of the recorded in-band radiance at select locations, i.e. the likelihood of the optical system detecting a certain radiance at any instance in time. Please note that this does not refer to the histogram of local radiation emission, but rather to the histogram of the radiation that has propagated through the flowfield and yields the projection of the plasma jet. The bottom row of inserted histograms corresponds to the centreline, while the top row shows a region near the edge of the plume. All histograms exhibit a Gaussian shape with varying width, which corresponds to the intensity value shown in the central heat map. This observation motivates the use of a Gaussian distributions for simulating random fluctuations in intensity for the remainder of this manuscript. Histograms of regions close to the edge exhibit a slightly skewed profile with a larger variance towards higher radiance values.

**Figure 10:**
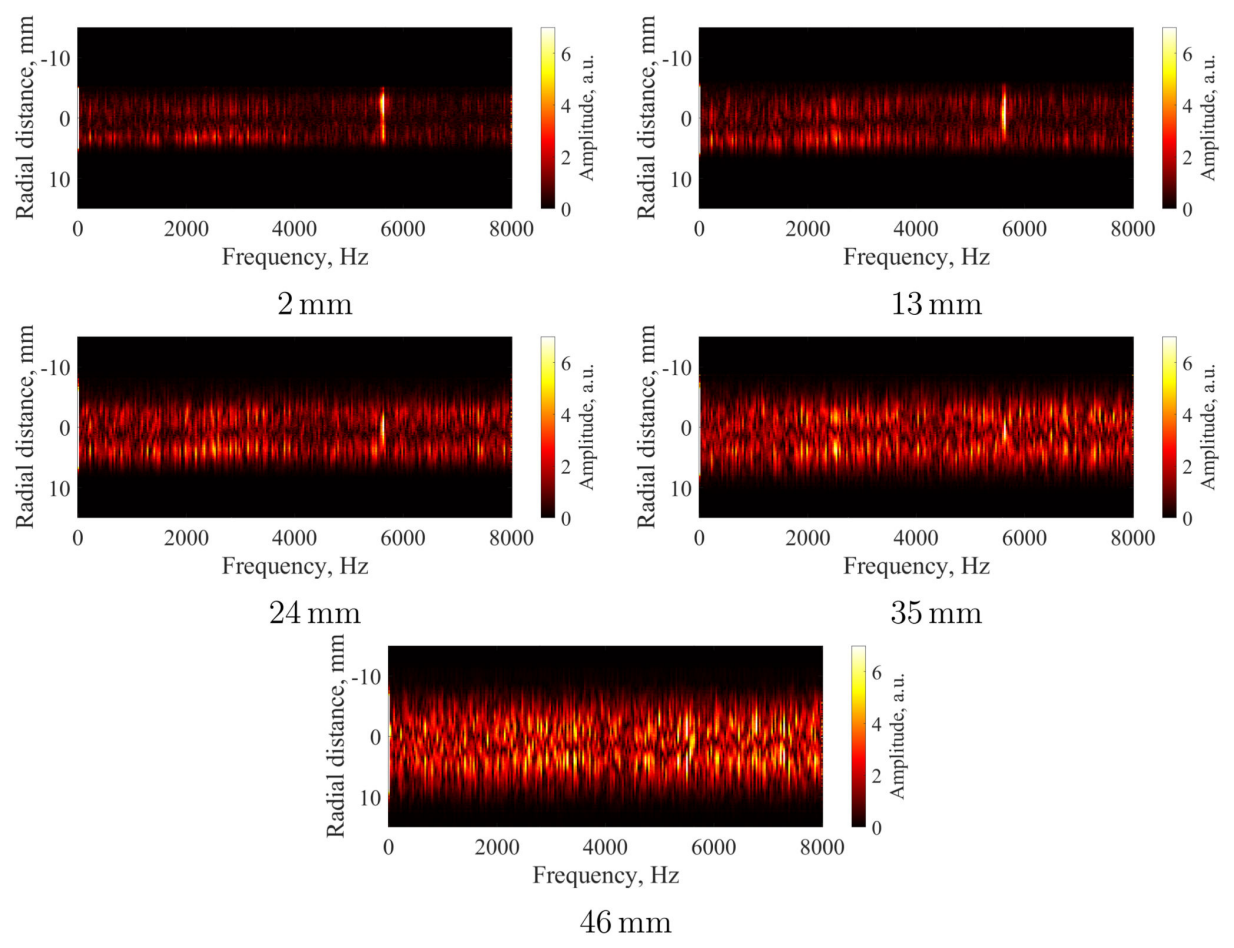
Radially resolved frequency spectra at 2, 13, 24, 35, and 46 mm from the nozzle exit plane.

A more detailed examination of the fluctuation frequency-spectra is carried out at the five individual cross-sections at 2, 13, 24, 35, and 46 mm from the nozzle exit plane. Figure 10 shows the discrete Fourier transform of the emitted radiation for each radial location of the plasma jet. A dramatic change in the spectra is observed as the plasma flow convects downstream. Close to the nozzle exit (2 mm), the spectrum is primarily influenced by a prominent single line at 5.6 kHz, which is likely associated with the power supply ripple frequency. This line is dominant across all radial locations, while a considerably weaker broadband spectrum appears in the background. The broadband contribution is relatively weak on the centreline and peaks in intensity along the edge of the plume. The presence of the broadband spectral trace and its concentration near the edge suggests that these fluctuations have originated from turbulent mixing at the external shear layer of the jet. As the flow convects downstream to larger distances from the nozzle, three effects are observed: i.) The intensity of the discrete mode at 5.6 kHz is diminishing and is concentrating more towards the centreline, ii.) the radial extent of the broadband spectrum is expanding and is becoming more homogeneous, iii.) the intensity of the broadband fluctuations is increasing. Effect i.) is likely due to fluctuations and mixing in flow direction. As the jet develops, the shear layer at the plume edge is continuously dissipating energy through turbulent mixing, i.e. packets of hot flow are mixed with colder fluid from the surrounding gas. This process leads to a spatial smearing of each pocket of gas. Unlike the discrete mode of the plasma leaving the nozzle, these turbulent processes span a wide range of frequencies [31]. As such, the discrete mode of 5.6 kHz is dissipated in regions of strong turbulent mixing and hence diminishes towards the edge of the plume. As the turbulent mixing intensifies in the downstream direction of the flow, the discrete mode diminishes progressively. Effect ii.) is consistent with previous findings, indicating that the plume is expansing in size towards a fully developed free jet profile [25]. The homogenisation of the spatial frequency content shows that the described mixing processes reach further into the centreline as the shear-layer grows downstream and eventually envelops the whole jet. Finally, effect iii.) shows that the strength of the fluctuations increases as the turbulent eddies become larger and contain more energy. This final effect can be intuitively inferred from some of the example images shown in Figure 5, e.g. the change in flow structure between 0 *µ*s, 62.5 *µ*s, and 125 *µ*s.

## 4 Analysis Methodology

This section provides a summary of the methods used to analyse the data and is divided into two subsections. The first section assumes quasi-steady state, and the second section expands the theory to account for unsteady flow effects.

### 4.1 Quasi-steady flows

This section employs established methods for steady or quasi-steady plasma plumes to determine an apparent radial temperature profile. As such, the results will build the baseline for an analysis in which the temporally averaged radiation is used and unsteady effects are neglected. The empirical radial enthalpy profile from the work of Fasoulas [25] is used according to

(4) $$h(r)=h_{\text{max}}{\left(1-{\left(\frac{r}{R_{s}}\right)}^{\frac{3}{2}}\right)}^{3}+h_{\infty }$$

with *h*(*r*) denoting the local mass-specific enthalpy at radius *r*, and *h*_∞_ denoting ambient local mass-specific enthalpy at the edge of the jet. *h*_max_ corresponds to the maximum local mass-specific enthalpy in the centre of the jet, and *R*_*s*_ corresponds to the jet width. Using NASA’s Chemical Equilibrium and Applications (CEA) program, these enthalpy values are converted into temperature under the assumption of chemical equilibrium [32]. Following this, the radial Argon total number density *n*_tot_ is calculated via the ideal gas equation

(5) $$n_{\text{tot}}(r)=\frac{p}{k_{B}T(r)},$$

with Boltzmann’s constant *k*_*B*_ and assuming a constant static pressure *p*, a common assumption for plasma free-jets [33]. The excited state number density *n*_*u*_ for the two Argon transition lines at *λ*_1_ =696.54 nm and *λ*_2_ =706.72 nm is determined assuming the Boltzmann distribution

(6) $$\frac{n_{u,1,2}(r)}{g_{u}}=\frac{n_{\text{tot}}(r)}{Q(r)}\text{exp}\left(\frac{-E_{u,1,2}}{k_{B}T(r)}\right),$$

with the upper state degeneracy *g*_*u*_, the partition function *Q*, and the upper state energy *E*_*u*_ [17]. NASA’s CEA tool and Eq.(6) are used under the assumption of local thermodynamic equilibrium. In this case, it is implied that the rate of chemical reactions, e.g. ionisation, is much faster than the local flow speed leading to a thermochemical equilibrium at every location within the flowfield [34]. It is further assumed that all excited atomic states are populated according to the Boltzmann distribution as is the case for collision-dominated plasmas [35]. The local volumetric emission coefficient *ϵ* for each line is calculated assuming spontaneous emission via

(7) $$\epsilon_{1,2}(r)=\frac{A_{ul,1,2}n_{u,1,2}(r)hc}{4\pi \lambda_{1,2}}$$

with the Einstein coefficient *A*_*ul*_, Planck’s constant *h* and the vacuum speed of light *c* [17]. These two emission signals are added to yield the total volumetric emission coefficient transmitted by the optical filter. Finally, assuming an optically thin and radially symmetric jet, the Radon transform [36] of the volumetric emission coefficient is taken to form a projected image of the plasma plume radiation. The resulting radiance signal can be directly compared to the calibrated high speed imaging data. In this procedure, the maximum temperature *T*_max_ and the jet radius *R*_*s*_ are varied until a best fit is obtained between simulation result and experimental data. This procedure is analogous to the methodology described in Ref. [20], albeit without the complication of additional chemical species.

### 4.2 Unsteady flow

In the case of unsteady flow behaviour, the local temperature will be a subject to random fluctuations *T*′(*x, y, z, t*) which will both be a function of time (*t*), axial (*x*), horizontal (*y*) and vertical (*z*) location. This leads to the approach of describing the instantaneous temperature as

(8) $$T(x,y,z,t)=\bar{T}(x,r)+T^{\prime }(x,y,z,t),$$

with the Reynolds averaged temperature $\bar{T}(x,r)$ which leads to the requirements of

(9) $$\frac{1}{\tau }\int_{0}^{\tau }T(x,y,z,t)\text{d}t=\bar{T}(x,r),\;\text{or},\;\frac{1}{\tau }\int_{0}^{\tau }T^{\prime }(x,y,z,t)\text{d}t=0,$$

where *τ* is a comparatively long time over which fluctuations occur sufficiently often to accurately represent their statistical distribution [37]. As was shown in Refs. [12, 21] arc-jet plumes can consist of many smaller random jets leading to flow structures which are highly non-uniform and not axisymmetric. However, over long times the statistical distribution of these events assumes an axisymmetric shape. This is also the case for the current investigation, as is shown by the symmetry of the jet characteristics, such as average radiance and standard deviation (see section 3). Hence, even though individual events are non-uniform and a function of *x, y, z*, their statistical occurrence assumes a uniform shape and can be described through an axisymmetric model which is only a function of axial (*x*) and radial (*r*) location. Therefore, the instantaneous temperature will be expressed through *T* (*x, r, t*) going forwards. This assumes an axisymmetric profile which is inaccurate when only a single instant in time is considered, but will be shown to represent the statistical average over long times.

**Figure 11:**
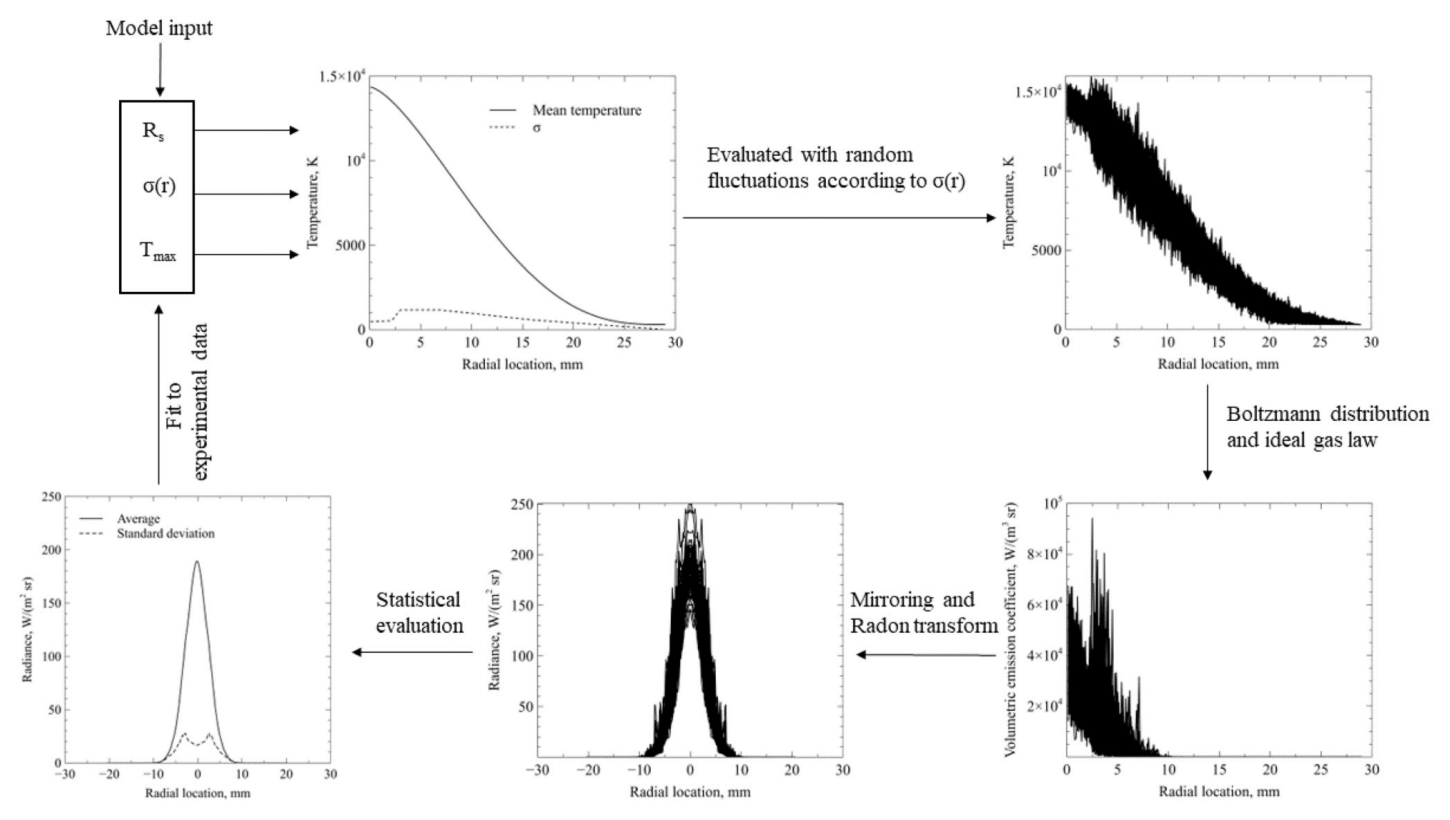
Flowchart of fitting algorithm for unsteady flow analysis.

As is shown in Figure 8, the probability distribution function of the projected radiance follows a shape similar to a Gaussian distribution. This motivates the use of a Gaussian distribution to describe the probability of local fluctuations in temperature according to the equation

(10) $$G(v)=\frac{1}{\sigma \sqrt{2\pi }}\text{exp}\left(-\frac{1}{2}{\left(\frac{(v-\mu }{\sigma }\right)}^{2}\right),$$

evaluated at *v*, with the standard deviation *σ*, and the mean value *µ* [38]. As the standard deviation of fluctuating radiance was shown to be axisymmetric in Figure 8, the instantaneous temperature *T* (*x, r, t*) is evaluated using a random fluctuation value that is assigned using Eq.(10) as the probability density function, with *σ*(*x, r*) and a mean value of $\mu (x,r)=\bar{T}(x,r)$. $\bar{T}(x,r)$ is described through the turbulent free-jet profile specified in Eq.(4). The specified procedure is performed for a large set of random numbers resulting in an equal set of instantaneous temperature profiles. Due to the conditions in Eq.(9), the temporal mean of the simulated temperature profiles recovers Eq.(4), as would be suggested by theory [25]. The following steps are carried out according to Eqs.(5-7) and the subsequent Radon transform to obtain a spatially resolved radiance profile. This procedure is performed for a large set of data spanning various random temperature profiles and the average and standard deviation radiance are computed for each location. Similar to the procedure laid out in section 4.1, the model input parameters *T*_max_ and *R*_*s*_ are varied until a match between simulation and experiment is obtained. The unsteady treatment of this procedure leads to the additional unknown of the enthalpy standard deviation distribution *σ*(*x, r*), that needs to be generated such that it satisfies the measured radiance standard deviation and the respective measured radiance histograms. In conclusion, this model requires a fit of both the average radiance profile and the standard deviation radiance profile. For this purpose, *σ*(*x, r*) is modelled as a linear interpolation between six individual points along the radial direction. The exact location and magnitude of these points is generated by a best fit algorithm that achieves the closest match between simulation and experiment for radiance standard deviation. The fitting loop is schematically depicted in Figure 11.

One noteworthy difference between this approach and the effects observed in experiment is that each single simulated profile is axisymmetric, whereas each instantaneous event in the experiment is not. As the simulations are carried out over a large number of individual distributions, the statistical properties of both experiment and simulation have to match. As such, the probability distribution of measuring the plasma radiance at a given location is approximately equal for experiment and simulation. Furthermore, as the plasma is assumed to be optically thin, the employed model does not lead to loss or trapping of energy due to non-linear self-absorption. Therefore, the total radiance emitted over long times remains the same in experiment and simulation.

## 5 Analysis Results

The methods in section 4 are used to investigate the plasma temperature distribution. The results are shown for the cases of assuming quasi-steady or unsteady behaviour respectively. The section further provides details of the unsteady flow characteristics and shows the measurement errors introduced by neglecting unsteady effects in the post-processing analysis.

### 5.1 Quasi-steady analysis

**Figure 12:**
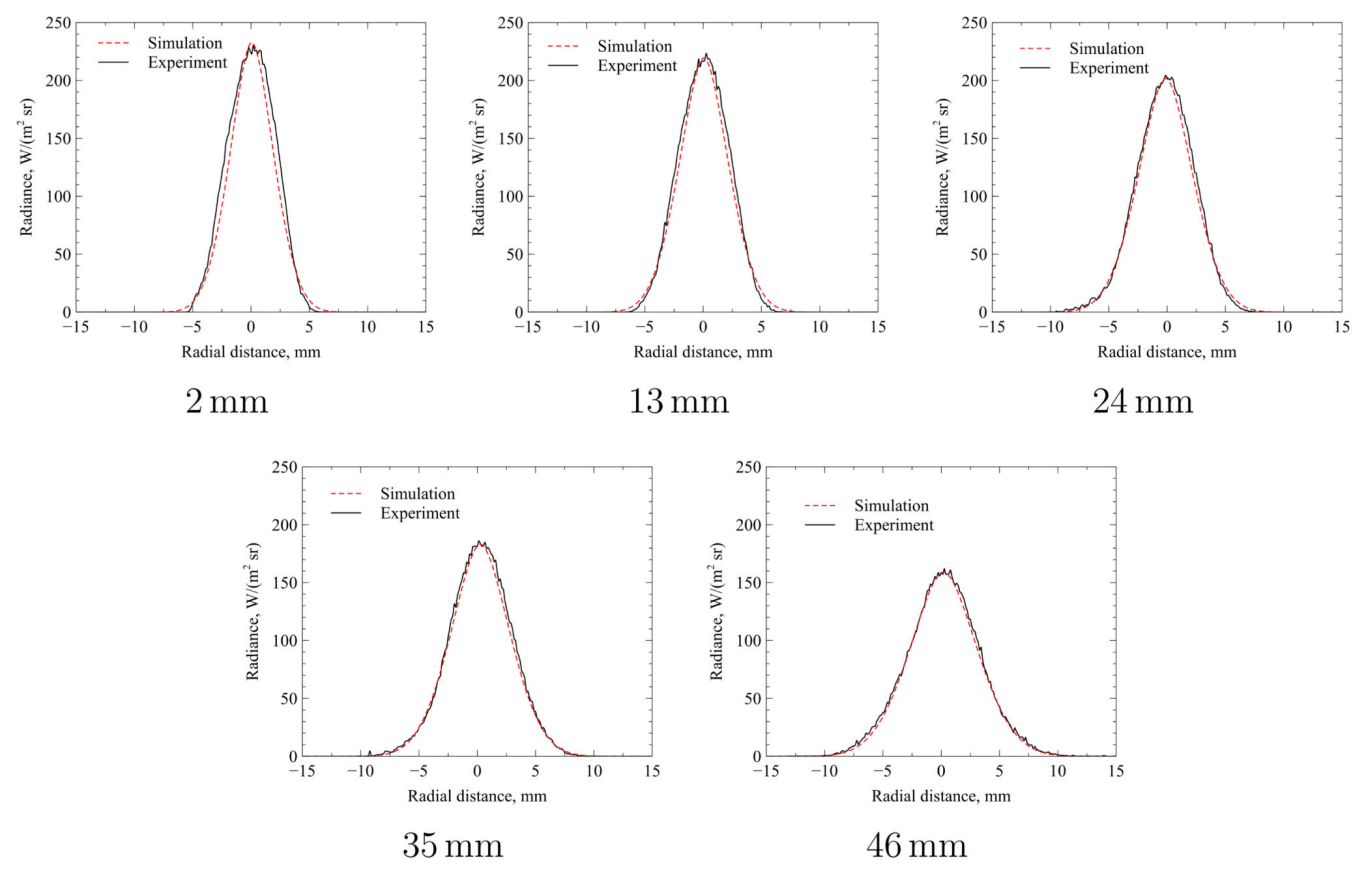
Comparison between radiance profiles at different axial locations using quasi-steady analysis.

The resulting plume radii and maximum temperatures are summarised in Table 1 and the fits for each cross section are shown in Fig. 12. As expected, the plume increases linearly in diameter as the flow convects downstream and the edge shear layer grows consistent with the observations from the literature [25, 37]. Since the total jet power remains constant or decrease through radiation losses, the maximum local mass-specific enthalpy on the jet centreline also decreases. As the jet convects downstream, the mass-specific enthalpy is spread out in radial direction and the maximum temperature decreases, as expected. The full radius of the temperature profile is significantly broader than the apparent radiation signature. This is because the intensity of emitted radiation diminishes rapidly as the temperature decreases in radial direction, i.e. gas that is not part of the hot core of the flow produces a negligible radiative emission signature. This is due to the strong non-linearity expressed in Eq.(6) which produces very few excited particles for lower temperatures. The data presented in Fig. 12 shows that the temporally averaged radiation profile of the experimental plume is approximately symmetric. The fit quality between simulation and experiment is excellent at larger distances, e.g. at 35 mm and 46 mm, but exhibits more discrepancies as the plane moves closer to the nozzle exit. The reason for this decline in fit quality is due to the deviation from self-similarity in this region [31]. The applied theory of Fasoulas [25] in Eq.(4) assumes self-similar flow. However, close to the nozzle the initial shear layer is developing from a nozzle exit flow profile towards a self-similar profile. Nevertheless, the fit quality is acceptable and the resulting enthalpy and radii will be used in the following discussion. Accounting for uncertainty in the model input parameters of measured counts per pixel and pressure, the derived temperature uncertainty

(11) $$\text{Δ}T=\sqrt{{\left(\frac{\partial T}{\partial p}\text{Δ}p\right)}^{2}+{\left(\frac{\partial T}{\partial n_{\text{exp}}}\text{Δ}n_{\text{exp}}\right)}^{2}}$$

is calculated according to the methodology of Moffat [39]. The partial derivatives in Eq.(11) are determined numerically using the evaluation methodology of section 4. The uncertainty of pressure measurement Δ*p* is based on the manufacturer accuracy of the pressure gauge, while the uncertainty in measured counts Δ*n*_exp_ is based on the shot-noise described in section 2.2. The overall uncertainty Δ*T* is found to be approximately *±*250 K.

### 5.2 Unsteady analysis

**Figure 13:**
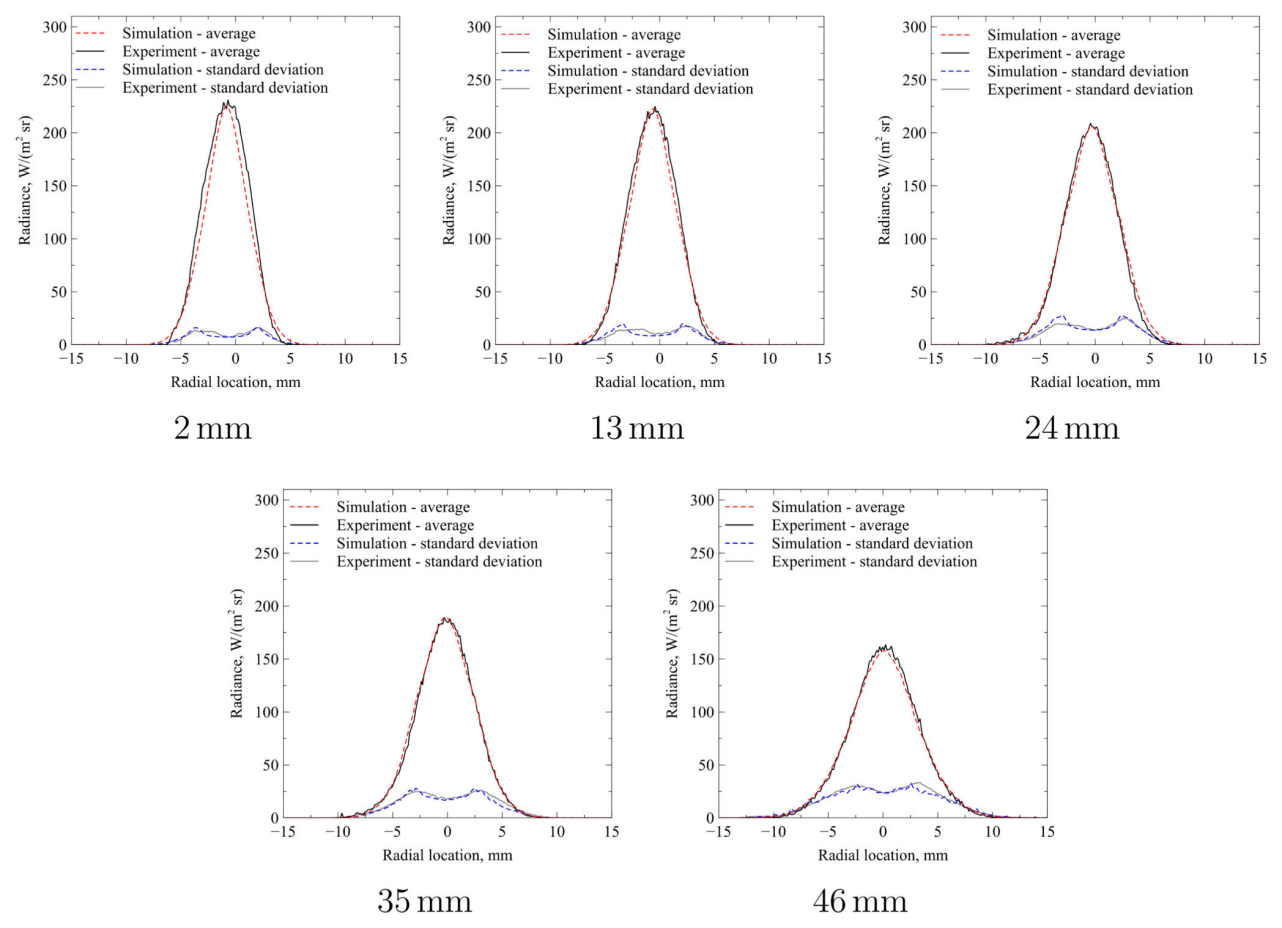
Comparison between radiance profiles at different axial locations using unsteady analysis.

Table 2 gives an overview of the fitted values for maximum temperature, jet width and maximum standard deviation. Please note, that the radial distribution of the standard deviation is also fitted individually for each cross section shown. The maximum values of that distribution are presented here to give a shorthand metric of the strength of fluctuation at each cross section. The fit of both the average and standard deviation radiance for each case are shown in Figure 13. A similar quality of fit is achieved as for the steady analysis in the preceding sub-section. The measured standard deviation exhibits a slightly asymmetric profile with a larger peak towards positive radial values. However, this deviation from axial symmetry is comparatively small.

For the first cross-section at an axial location of 2 mm, the values found are almost identical with the respective fit using a quasi-steady analysis. This is unsurprising, as the relative magnitude of fluctuations is small at locations close to the nozzle exit. In this region, the free-jet is in its developing regime and only a small portion of it has been affected by the external shear layer. The expected trend found in the quasi-steady analysis for falling maximum temperature and increasing jet width is also confirmed here [25, 37]. However, some notable differences exist: The maximum temperature is smaller if the cross-sections are evaluated by accounting for unsteadiness. Furthermore, the jet width is smaller. Both effects are due to the non-linear emission of radiation where high temperature fluctuations artificially broaden the jet and increase it’s apparent peak temperature value. This is due to the fact that the radiative projection of the jet is dominated by fluctuations producing local instantaneous temperatures higher than the temporal mean. As the respective radiative emission is non-linear and grows exponentially with temperature, these high-temperature events tend to skew the average radiance towards higher values. By accounting for unsteadiness in the flow, the bias introduced by these effects is corrected.

## 6 Discussion

**Figure 14:**
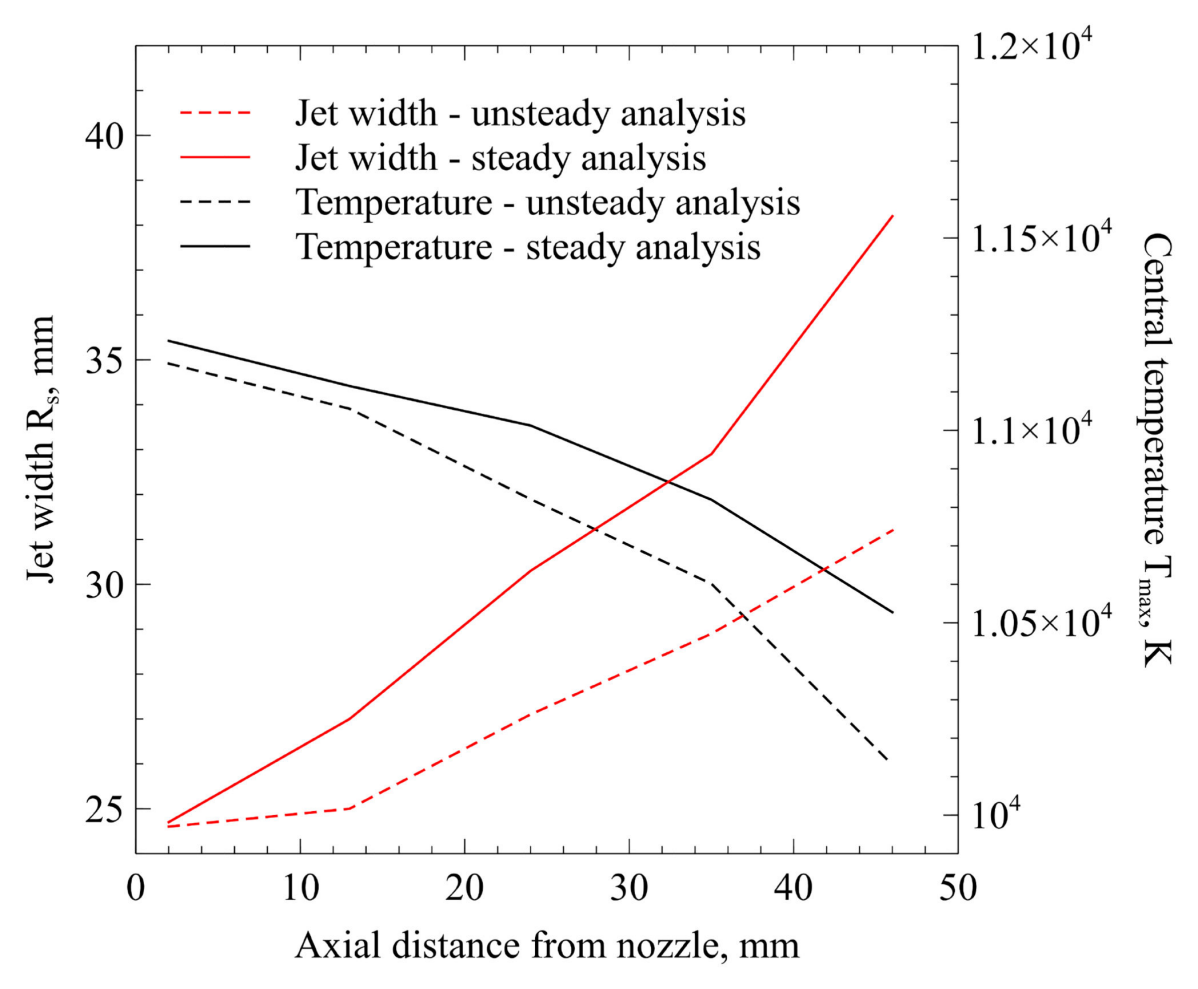
Central temperatures and jet widths along the axial direction of the flow.

This section discusses the obtained measurement outcomes and gives additional justification to the analysis method by considering the momentum and energy conservation of the plasma jet. Figure 14 displays the obtained jet widths and central temperatures for both types of analysis. It is clear that accounting for unsteady effects leads to a slightly lower peak temperature and to significantly smaller jet diameters. Furthermore, the rate of growth in jet diameter based on unsteady analysis is slower than for the quasi-steady analysis. This is due to the larger scale of fluctuations at increasing axial location. In the regions where fluctuations are comparatively small, i.e. near the nozzle exit, the agreement between both methods is good. However, as the jet eddies become larger and lead to stronger fluctuations compared to the average radiance, the error introduced by these unsteady effects becomes larger. Consequently, the plasma plume appears erroneously large. Figure 14 clearly shows that analysis neglecting unsteady effects will suffer from a measurement bias that artificially increases temperature and jet width.

**Figure 15:**
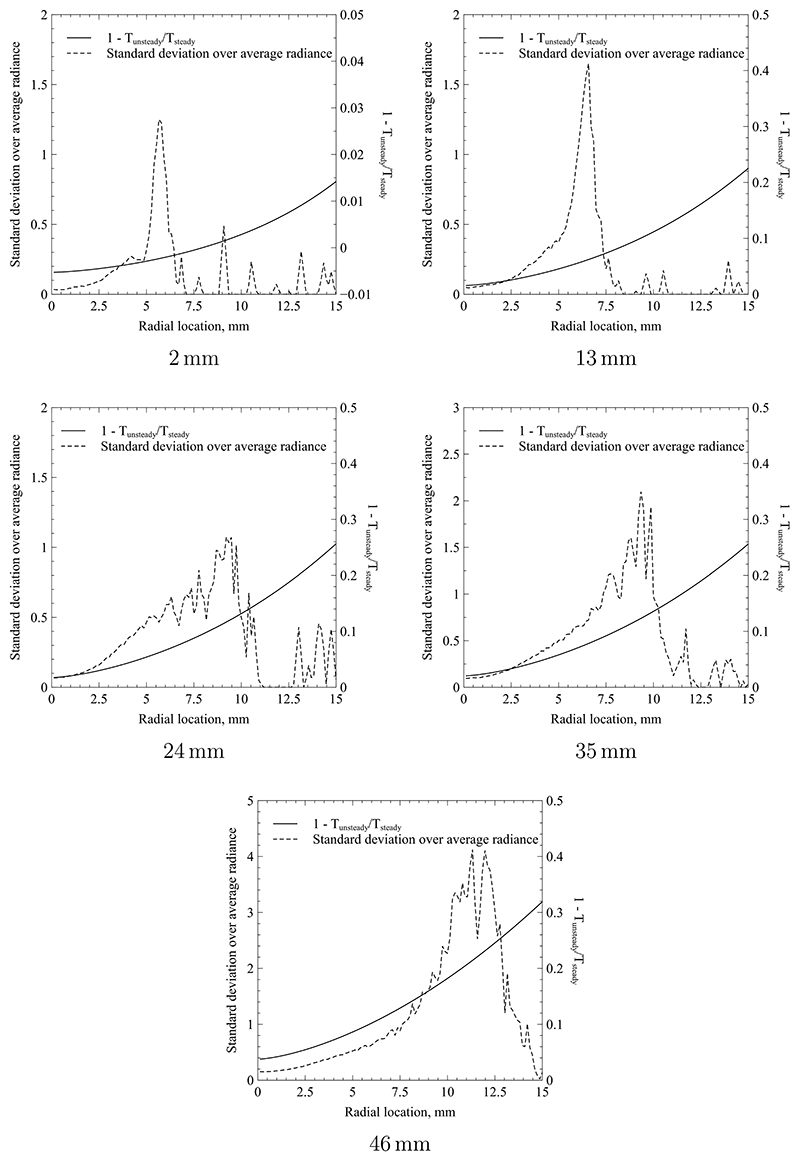
Difference in temperature determination due to neglecting unsteady effects in the plasma jet.

The radial difference in obtained temperature based on the two methodologies is shown in Figure 15 for each investigated cross-section. The normalised difference is expressed as

(12) $$\text{Δ}=1-\frac{T_{\text{unsteady}\;}}{T_{\text{steady}}}$$

giving a relative bias when neglecting unsteady effects. For all cross sections, the agreement between quasi-steady and unsteady cases is best on the centreline and in the region of 0.1% to 4% with the best match close being to the nozzle. However, the difference quickly rises with increasing radius reaching up to 32% for the most downstream cross-section. The difference continues to rise beyond the maximum radius shown. However, only values up to 15 mm are presented as distances further away do not produce a measurable emission signal and are hence extrapolations using the self-similar temperature profile used in Eq.(4). The figures also contain the ratio of standard deviation over average radiance, which can be interpreted as the relative unsteadiness of the projected radiation. The temperature error somewhat follows the trend of this ratio until the peak which occurs near the jet edge. The magnitude of the difference also correlates with the magnitude of the measured relative radiance fluctuations. However, no direct relationship can be drawn between the two curves shown, as the radiance ratio is the result of an integral transformation, i.e. the Radon transform, and therefore looses its direct link to a specific radial location. The analysis shows that the measurement bias introduced by neglecting unsteady effects leads to too high temperatures near the edges of the plume. This implies that a quasi-steady analysis overestimates the enthalpy convected near the jet edges which will be shown to be erroneous in the following discussion.

Both the increased temperature and radial extent obtained from quasi-steady analysis lead to a measured total plume power that is too large, which is detailed in the following. The increased jet diameter implies a larger mass flow rate within the flow, e.g. more entrained fluid. The higher peak temperature implies a larger enthalpy of this transported fluid. Those two aspects are evaluated by considering the momentum and energy conservation within the plume. Based on the model of a turbulent Schlichting jet [37], the energy and momentum of the jet are constant,

(13) $$E=2\pi \int_{0}^{\infty }\rho \bar{u}\left(\bar{h}-h_{\infty }\right)r\;\text{d}r=\;\text{const}.\;$$

and

(14) $$K=2\pi \int_{0}^{\infty }\bar{\rho }{\bar{u}}^{2}r\;\text{d}r=\;\text{const}.$$

with Reynolds averaged velocity $\bar{u}$, density $\bar{\rho }$ and enthalpy $\bar{h}$, and ambient enthalpy *h*_∞_. Please note that the equations given in Ref. [37] have been extended by the density term in order to account for compressibility in the plasma jet. Consistent with Fasoulas’ model used in Eq.(4),

(15) $$\bar{u}(r)=u_{\max}{\left(1-{\left(\frac{r}{R_{s}}\right)}^{\frac{3}{2}}\right)}^{2},$$

is used to describe the mean velocity distribution [25]. The maximum velocity *u*_max_ present on the symmetry line of the flow is determined by considering both the conservation of mass and momentum. The value immediately outside the nozzle exit plane is calculated by considering the measured thermodynamic properties at the cross section *x* = 2 mm and assuming no further entrained flow. This leads to the requirement

(16) $$2\pi \int_{0}^{\infty }\bar{\rho }\bar{u}r\;\text{d}r={\dot{m}}_{\text{nom}},$$

with the measured nominal mass flow rate ${\dot{m}}_{\text{nom}}$. of Argon entering the plasma generator [37]. The density at each radial location is determined through the look-up tables provided in NASA’s Chemical Equilibrium and Applications (CEA) program [32], using the measured test section pressure and the temperatures determined through analysis of section 5. This allows the combination of Eqs.(15) and (16) to find the value for *u*_max_. In subsequent cross-sections downstream, the total mass transported by the jet has increased due to flow entrainment in the external shear layer. However, as described in Eq.(14), the total momentum *K* has to remain constant. Hence, the velocity profiles, i. e. *u*_max_, at each cross-section need to be scaled such that Eq.(14) is satisfied. This procedure assumes a self-similar jet that conserves its momentum [37].

**Figure 16:**
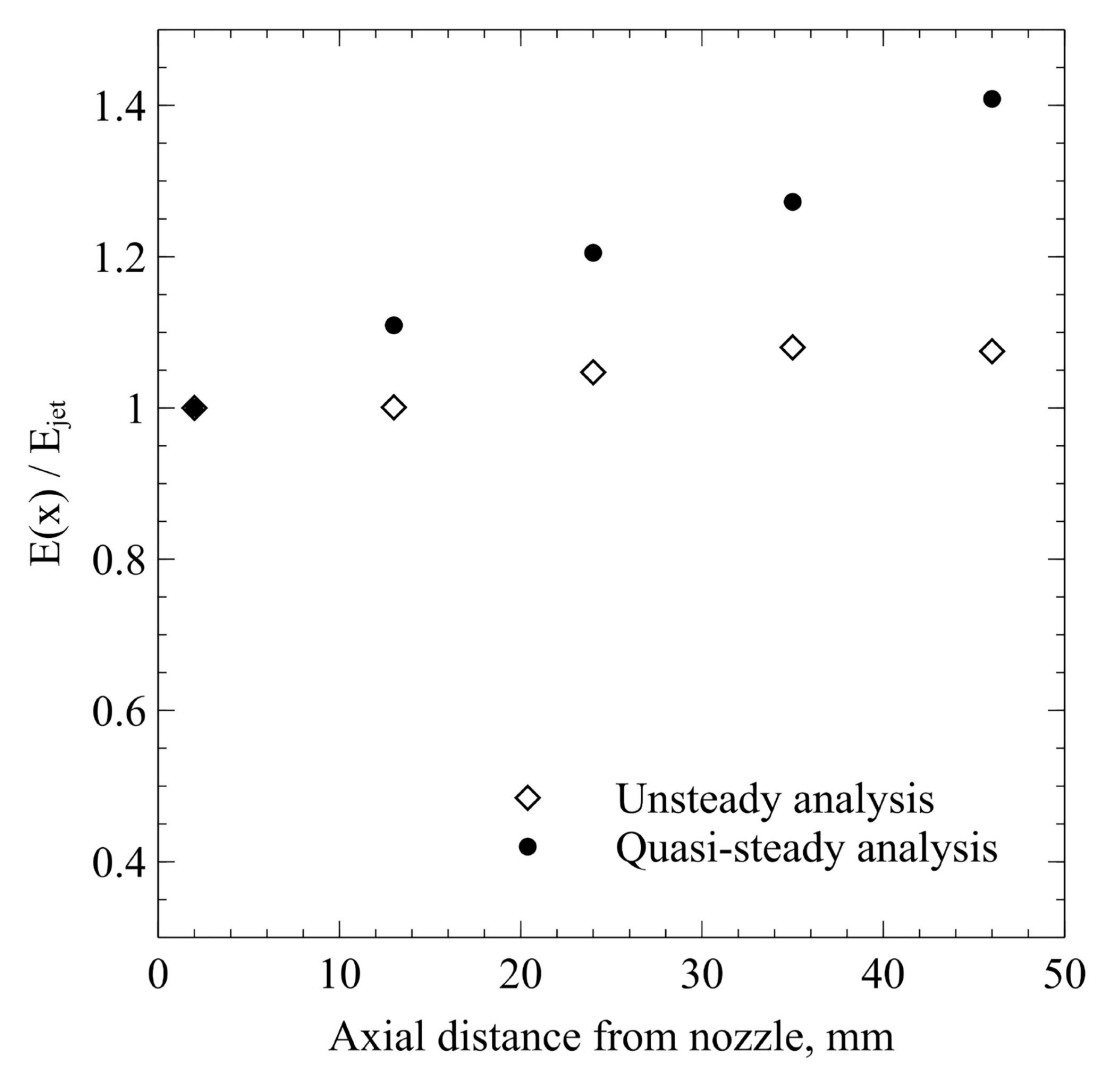
Ratio of integral jet energy at location *x* over total jet energy at nozzle exit.

The analysis is carried out for both the results obtained with quasi-steady and unsteady assumptions. The two respective datasets are then tested using Eqs.(13) which describes the conservation of energy. Based on classical fluid mechanics analysis the total jet power needs to stay constant. In the present case, some radiation losses are expected which will continuously lower the total jet power as it convects downstream. However, these losses are significantly lower than the convective energy content of the flow. Hence, Eq.(13) is utilised at each investigated cross-section. The mean enthalpy $\bar{h}$ is calculated through CEA’s look up tables based on the measured test section pressure and the temperatures obtained from the aforementioned analysis methods [32]. The resulting total plume power is plotted in Figure 16 normalised by the power obtained in the first measured cross-section at *x* = 2 mm (*E*_jet_). The unsteady analysis shows a very slight rise of *E*(*x*) up to approximately 6% which is assumed to be within the uncertainty margin of the measurements. This demonstrates that the current unsteady analysis satisfies both the momentum and energy conservation at the same time. In contrast, the results derived from the quasi-steady analysis exhibit a continuous increase of the total jet power surpassing the initial value by up to 41%, which clearly contradicts the first law of thermodynamics. This suggests that the measured temperature and plume widths under quasi-steady assumptions overestimate enthalpy content in the plasma flow significantly.

## 7 Conclusion

This work describes measurements of an unsteady Argon plasma free jet in the miniaturised arc-jet facility OPG1. Spectrally narrow bandpass filtered images of the plasma radiation were captured using a high speed camera and have been calibrated for absolute radiance using an integrating sphere. The spectrally integrated plasma emission from the two Argon atomic transition lines at 696.54 nm and 706.72 nm was captured from the nozzle exit plane up to 46 mm downstream. The resulting plasma free jet radiance was found to be axisymmetric both in average radiance and standard deviation. Fluctuations were shown to exhibit a close to Gaussian probability distribution function at various locations of the radiation field. Frequency analysis revealed that the temporal plasma fluctuations transition from a single frequency at 5.6 kHz to a broadband spectrum as the jet convects downstream and the turbulent mixing processes become stronger.

The spatially resolved temperature was determined by fitting a theoretical model to the measured radiance, using either quasi-steady or unsteady flow physics. It was observed that the assumption of a quasi-steady plasma flow leads to a significant overestimation of both temperature and jet width. The steady and unsteady models agree well directly at the nozzle exit where only small fluctuations exist and the jet shear layer is narrow. As the flow develops, fluctuations and shear layer grow in size and the models deviate significantly from each other. 46 mm from the nozzle plane, the steady analysis overestimates the jet width by 18%, the central temperature by 4% and the radial edge temperature by 32%. This results in an overestimation of as much as 41% power contained within the free jet. The current work utilises absolute radiance measurements to infer temperature, but the present findings are similarly applicable to other spectroscopic methods relying on measured radiative emission, such as Boltzmann plots, spectral band fits or two-line ratios. Even though the other mentioned methods can be carried out using normalised radiance measurements, the relative line intensity will be dominated by fluctuations above the mean plasma temperature.

The present work has demonstrated that achieving an accurate optical characterisation of unsteady plasma free jets is only feasible, if the temporal fluctuations of temperature are considered. While the centreline results are less affected by this issue, the measurement bias of assuming steady behaviour is significant for locations further away from the axis of symmetry.

## Figures and Tables

**Figure 4:**
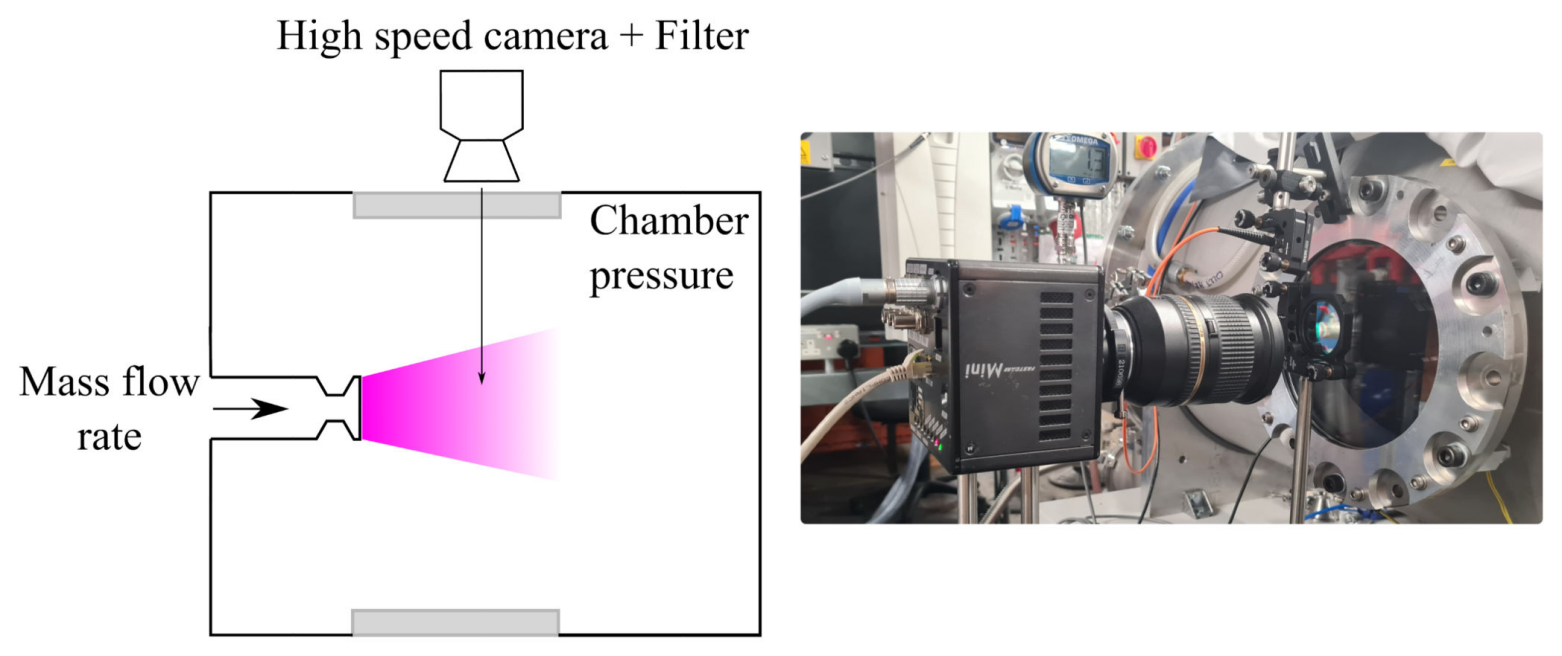
Schematic of imaging setup (left) and photo (right).

**Table 1:** Plume characteristics obtained from quasi-steady analysis.

*x*, mm	*T*_max_, K	*R_s_*, mm
2	11,233	24.7
13	11,115	27.0
24	11,013	30.3
35	10,820	32.9
46	10,528	38.2

**Table 2:** Plume characteristics obtained from unsteady analysis.

*x*, mm	*T*_max_, K	*σ*_*h*,max_, K	*R_s_*, mm
2	11,174	545	24.6
13	11,056	559	25.0
24	10,821	809	27.1
35	10,601	853	28.9
46	10,131	1,294	31.2
